# Examining concussions in adult male, senior-grade semi-elite rugby league in Australia: A retrospective observational video review case series

**DOI:** 10.1016/j.jsampl.2024.100086

**Published:** 2025-01-02

**Authors:** Martin A. Lang, Grant L. Iverson, Suzi Edwards, Ben Jones, Douglas P. Terry, Andrew J. Gardner

**Affiliations:** a School of Medicine and Public Health, College of Health, Medicine and Wellbeing, University of Newcastle, Callaghan, New South Wales, Australia; b Department of Physical Medicine and Rehabilitation, Harvard Medical School, Boston, MA, USA; c Department of Physical Medicine and Rehabilitation, Spaulding Rehabilitation Hospital, Charlestown, MA, USA; d Department of Physical Medicine and Rehabilitation, Schoen Adams Research Institute at Spaulding Rehabilitation, Charlestown, MA, USA; e Mass General Hospital for Children Sports Concussion Program, Boston, MA, USA; f Sydney School of Health Sciences, The University of Sydney, Camperdown, New South Wales, Australia; g Carnegie Applied Rugby Research (CARR) Centre, Carnegie School of Sport, Leeds Beckett University, Leeds, United Kingdom; h Department of Neurological Surgery, Vanderbilt University School of Medicine, Nashville, TN, USA; i Vanderbilt University Medical Center, Nashville, TN, USA; j Vanderbilt Sports Concussion Center, Nashville, TN, USA; k Center for Cognitive Neurosurgical Studies, Nashville, TN, USA

**Keywords:** Rugby league, Concussion, Head injuries, Injury surveillance

## Abstract

**Background:**

The risk of concussion at the elite level of rugby league has been extensively evaluated. However, there has been very little concussion research conducted at the semi-elite level.

**Purpose:**

To examine cases of medically diagnosed concussion from a single season of adult men's semi-elite rugby league.

**Methods:**

A retrospective review of the 2019 Queensland Cup season head injury assessment surveillance program was completed. All Head Injury Assessment (HIA) cases, including cases of medically diagnosed concussion were retrospectively video reviewed and game play characteristic variables along with video signs of concussion were coded. This data was combined with the return to play data to form the research database.

**Results:**

There were 132 players removed for HIAs in 170 games. There were 36 players medically diagnosed with concussions, which equates to an incidence rate was 6.11 concussions per 1000 player match hours, or one concussion every 4.7 matches. All concussions occurred in a tackle event, where the player was struck in the head/face. Possible balance disturbance was the most commonly observed video sign (97.2 ​%; 35/36), with slow to stand also commonly observed in concussed players (91.7 ​%; 33/36). Most concussed players (63.9 ​%; 23/36) did not miss a game following the concussion.

**Conclusion:**

This is one of the first studies to review video footage of concussions in sub-elite rugby league. These findings build on the growing body of video analysis research in rugby league and suggest that the retrospective review of the video of incidents may offer insights into modifiable risk factors that may help reduce concussion in rugby league.

**Key points**

- The concussion incidence rate in men's sub-elite level rugby league is lower than the concussion incidence rate previously reported in men's professional level rugby league.
- Tacklers sustained more concussions than ball carriers and upright tacklers with an upright ball carrier accounted for 61.1 ​% (22/36) of concussions.
- Possible balance disturbance was the most commonly observed sign on retrospective video review of players medically diagnosed with concussed.
- Approximately 64 ​% of the male sub-elite level rugby league players did not miss a game following a medically diagnosed concussion.

## 1 Background

Rugby league is a full-contact sport involving numerous collisions and tackles. Rugby league match play is continuous, over two 40-min halves, with the same players involved in both offensive and defensive plays. The National Rugby League (NRL) is the highest level of competition in Australia. On average, during the 2014 season, there were more than 600 tackles per match in the NRL [1]. During game play, tacklers are more likely than ball carriers to sustain a blow to the head leading to an off-field medical evaluation, and this is more likely when the tackler is upright compared to bent-at-the-waist [2]. Medically diagnosed concussions are more likely to occur during high tackles [3]. During the 2014 season of the NRL, there was approximately one concussion every 3.35 games [1], and a significantly greater proportion of concussions were diagnosed during the first match of the season compared to all the other matches [4]. During the 2017 and 2018 seasons, there was approximately one concussion every three games (i.e., one every 2.7 matches) [5], and across those two seasons, the median number of days until medical clearance was 6 (interquartile range ​= ​4–7 days; range ​= ​0–79 days), and most players (88 ​%) did not miss a game after injury [5]. The presence of video-identified possible brief loss of consciousness (LOC) [6] or motor incoordination upon standing [7] were not predictive of time to recovery following concussion in two studies with NRL players.

Researchers have examined concussions in the NRL using video analyses [1,3,8], injury surveillance data [5], and combinations of video analyses and medical evaluation data [6,7,9]. However, there is a paucity of research relating to the video analysis of concussion in the lower (sub-elite) leagues. In 2019 in Australia, there were over 160,000 registered male and female players nationally, with 62,009 of those registered players based in the state of Queensland [10].

The Queensland Cup (QLD Cup) is Queensland Rugby League's (QRL) highest level of men's club competition. The QRL is the governing body of sub-elite rugby league in the state of Queensland, Australia. The QLD Cup predominantly features younger (mostly aged 19–23 years), less experienced athletes and is the primary sub-elite competition pathway to the NRL. The purpose of this study is to (i) determine the single season incidence of concussion in the QLD Cup, (ii) examine the game-play characteristics associated with concussions, (iii) inspect the video signs of possible concussion, and (iv) document time to return to sports following injury.

## 2 Methods

### 2.1 Participants

All players competing in the 2019 QLD Cup men's competition were participants in the QRL head injury assessment surveillance research program. All players gave prior consent to the Rugby League Players Association and the QRL to have their deidentified data used in research endorsed by the QRL Research Committee. This study was approved by the governing institution human ethics committee and allowed by the QRL research committee. The footage for all 132 players removed for a HIA, including the 36 players medically diagnosed with concussion were reviewed and coded in this study.

The men's QLD Cup includes 14 teams, competing over 23 rounds, with a four-week finals system between eight qualifying teams. Therefore, during a regular season, there are 161 games (7 pairs of teams, each playing 23 games). The finals (post-regular season) feature 9 games making a total of 170 games in the season. A Head Injury Assessment (HIA) was identified as a head impact that necessitated either the temporary removal of a player who was suspected of having sustained a concussion, resulting in an off-field screening process, or the permanent removal of a player diagnosed with a confirmed concussion. The HIA process is also used across several leagues and sports (e.g., World Rugby [11], National Rugby League [12], Australian Football League [13]). The QRL provided the research team with access to the match footage and all de-identified head injury assessment data.

### 2.2 Video coding

The video analysis was conducted using the STATS Edge (Stats Perform, London, United Kingdom) platform, with full access provided by the QRL. The broadcasted games provided multiple camera angles, with one broadcasted game scheduled per week [23 of the 161 regular season games (∼14 ​%) and all nine of the finals series games; 32/170 or ∼19 ​% of games for the entire season]. For the non-broadcasted games, one camera angle was available.

The video analysis was conducted by a single author (MAL), using a coding matrix including 46 variables for each incident. Many of the variables that were coded have been described previously [14]. The coding matrix was developed using templates that have been applied to video analysis research in professional rugby union and rugby league [3,15]. Variables include game play characteristics (e.g., type of play, number of tacklers involved in the tackle, tackle number in the set, location on the field, etc.), tackle characteristics (e.g., tackle height, average speed of the players entering the tackle, ball carrier evasion technique, etc.), and video signs of potential concussion. Many variables are coded as present (yes/no), other variables are numerical (e.g., tackle number in the set), while others are coded describing the characteristic/variable (e.g., tackler body position: upright, bent at the waist, bent at the knees, diving, jumping etc.) by the video reviewer (i.e., the first author in this instance) and recorded in an excel spreadsheet. These variables have been previously described [16,17]. The variable *tackle number in the set* ranges from zero to six. Tackle zero, which may occur when a team knocks the ball on and the opposition gain possession of the ball, the initial tackle following the loss of possession is ‘tackle zero.’ In addition, when an attacking team kicks the ball dead or knocks the ball on over the tryline, the opposition restart the game from the 20 ​m line and the initial tackle in the set is ‘tackle zero.’ Essentially, the set becomes a seven tackle set, instead of a six tackle set. See the online supplementary material from the study by Gardner and colleagues published in 2021 for the definitions of these variables [17].

The video reviewer coded two groups of video signs. First, the six international consensus video signs of potential concussion [18]. Second, the video signs from the NRL's Category I and Category II signs of potential concussion [17]. These NRL Category I and II video signs include an additional three video signs (i.e., possible tonic posturing, possible balance disturbance, and slow to stand) that were evaluated together with the six consensus video signs, for a total of 9 video signs of potential concussion.

The video signs of potential concussion were operationally defined as: (i) *balance disturbance*: evidence of wobbly legs and being unable to walk normally, and/or upon standing, the player is unsteady on his/her feet, stumbles, falls, or is unable to walk without assistance; (ii) *seizure*: clonic movements or convulsions; (iii) *tonic posturing*: stiffening of the limbs; (iv) *no protective action*: does not have the ability or awareness to protect himself when falling to the playing surface; (v) *dazed/vacant/blank look*: the player is unable to visually focus or is looking toward the distance when being assessed or leaving the playing field; (vi) *lying motionless*: lies or remains motionless on the ground for a period of time longer than what would usually be deemed normal; (vii) *possible tonic posturing (from the NRL Category II video signs)*: possible stiffening of the limbs; (viii) *possible balance disturbance (from the NRL Category II video signs)*: possible or very mild unsteadiness upon standing; (ix) *slow to stand*: the player gains their feet at a slower rate that would be deemed normal. The difference between some Category I and Category II signs (tonic posturing vs. possible tonic posturing; balance disturbance vs. possible balance disturbance) relates to the certainty of observing the sign. If it was not definitive that tonic posturing and/or balance disturbance was observed, but there is suspicion that it may have been, then the use of the Category 2 sign ‘possible tonic posturing’ and/or ‘possible balance disturbance’ was applied. For clear and definitive cases, the sign is considered to be a Category 1 sign.

### 2.3 Video coding training

The first author (who is a former professional rugby league player and veteran of 176 first grade games at the elite level) was the main video reviewer/coder of cases. He was trained on the definition of each variable and how to apply the definition to each case by the senior author. The senior author has extensive experience coding video of tackle events, HIAs, and medically diagnosed concussions. As part of the training, the first and senior authors independently coded the first 25 cases in the database, and the senior author provided feedback and further training on any discrepant variables. Both authors then independently coded an additional 5 cases, with the agreement between the first and senior authors of these 5 cases being perfect (i.e., 100 ​%). The first author then independently completed the coding of all HIA cases.

### 2.4 Return to play

In the men's QLD Cup, the return to play process is managed by the team medical staff. As per the NRL Concussion Policy, players suspected of having sustained a concussion are evaluated and provided with a medical recommendation on the day of injury. A subsequent re-evaluation is conducted 48 ​h after injury. The graduated return to play process is completed in six stages. A player diagnosed with concussion may be medically cleared to return to contact training and match play by the club doctor. The player must be asymptomatic at rest and at each stage of the graduated return to play process. For this study, the number of games missed following the game that they sustained their concussion was gathered from online archived team sheets (https://www.qrl.com.au/draw/?competition=114&amp;round=1&amp;season=2019) [19], which were reviewed to record when a concussed player returned to play in a subsequent game, with the number of weeks to return to play recorded in the database.

### 2.5 Incidence of concussions

The incidence of concussions was calculated by dividing the number of concussions by the total number of matches in the season (n ​= ​170) multiplied by the number of players in the game (n ​= ​26) multiplied by the game duration (80mins, or 1.33 ​h). The result was then multiplied by 1000 to report the incidence of concussions per 1000 player hours. The average number of matches played for every 1 concussion was calculated by dividing the total number of matches in the season (n ​= ​170) by the total number of concussions.

## 3 Results

During the 2019 men's QRL season, there were 170 games and 132 players who underwent an HIA. There was a total of 36 medically diagnosed with concussions (36/132 ​= ​27 ​%), among 34 different players. Two players were diagnosed with two concussions during the season. The concussion incidence rate was 6.11 concussions per 1000 player match hours, which is equivalent to one concussion every 4.7 QRL matches.

### 3.1 Game-play characteristics

There were 34/36 (94.4 ​%) concussions that occurred in a tackle event. There were no tackles that resulted in more than one player being medically diagnosed with concussion, despite there being 13 (36.1 ​%) concussions that were a result of head-to-head contacts. All concussions resulted from players who were struck in the head/face. The mechanisms of contact are presented in Table 1 and Table 2. Approximately one in 10 (11.1 ​%) of the concussions were a result of foul play (4/36 cases).

**Table 1:** Characteristics of tackles involving concussions.

Characteristic	f	%
**Mechanism of head contact**		
Head-to-Head	13	36.1
Tackler-to-ball carrier	9	25.0
Tackler-to-tackler	4	11.1
Shoulder	6	16.7
Knee	2	5.6
Elbow	1	2.8
Forearm	4	11.1
Hip	1	2.8
Playing surface	4	11.1
Unknown	5	16.1
**Tackler position**		
Upright	25	69.4
Bent-at-Waist	4	11.1
Diving	3	8.3
Leaping/Jumping	3	8.3
Flopping/Tackling	1	2.8

There was a total of 36 tackles involving concussions. f ​= ​frequency and % ​= ​percentage.

**Table 2:** Frequency of concussion signs on video analysis.

Video Signs	f	%
Balance disturbance/Motor incoordination	26	72.2
Seizure	1	2.8
Tonic posturing	0	0.0
No protective action	13	36.1
Dazed/Blank stare/Vacant look	11	30.6
Lying motionless	8	22.2
Possible tonic posturing^a^	8	22.2
Possible balance disturbance^a^	35	97.2
Slow to stand^a^	33	91.7
No signs observed	2	5.6

Number of consensus video signs		

0	8	22.2
1	12	33.3
2	10	27.8
3	5	13.9
4	1	2.8
5	0	0.0
6	0	0.0

a The signs *Possible Tonic Posturing, Possible Balance Disturbance,* and *Slow to Stand*, were the three video signs of potential concussion that were not part of the six consensus video signs. These signs are Category 2 signs, which are used by medical staff to identify players who should be removed from play for a medical evaluation.

In terms of the playing positions (as determined by match reports), there were 25 concussions that occurred to forwards and 11 concussions that occurred to backs. There were 10 concussions that occurred to the ball carrier compared to 24 concussions to the tackler. In 2 concussion cases, a player was neither a ball carrier nor a tackler (one occurred as a late hit off the ball and the other was an aerial contest from a cross-field bomb). There were 9 concussions that occurred in one-on-one tackles, 15 concussions that occurred when there were two tacklers, and 12 concussions involved three tacklers.

Of the 24 tacklers who were diagnosed with a concussion, 12.5 ​% (3/24) occurred when the tackler was engaging in a low tackle, compared to 87.5 ​% (21/24) when the tackler was engaging in a high tackle (i.e., initial contact being made above the nipple line, to the shoulders, neck, or face/head). Of the 24 concussions that occurred to a tackler, 15 (62.5 ​%) were to the first tackler into the contact, 8 (33.3 ​%) to the second tackler into the tackle, and one (4.2 ​%) to the third tackler into the tackle. For the ball carriers who were diagnosed with a concussion, all 10 (100 ​%) occurred when the tackler was engaging in a high tackle.

In terms of the ball carrier body position, 83.3 ​% (30/36) of concussions occurred when the ball carrier was upright, 5.6 ​% (2/36) occurred when the ball carrier was bent-at-the-waist, 5.6 ​% (2/36) occurred when the ball carrier was falling/diving, 2.8 ​% (1/36) occurred when the ball carrier was on the ground, and 2.8 ​% (1/36) of the concussions occurred when the ball carrier was bent at the knees. An upright tackler and an upright ball carrier accounted for 61.1 ​% (22/36) of concussions.

There were 22 (61.1 ​%) concussions that occurred in the middle corridor of the field. There were 6 (16.7 ​%) concussions that occurred in the defensive quarter, 9 (25 ​%) in the midfield defensive quarter, 9 (25 ​%) in the midfield attacking quarter, and 12 (33.3 ​%) in the attacking quarter.

There were 9 (25 ​%) concussions that occurred in the first half and 27 (75 ​%) concussions that occurred in the second half. Dividing the game into 20-min quarters, there were 3 (8.3 ​%) concussions that occurred in the first 20 ​min, 6 (16.7 ​%) between 21 and 40 ​min, 6 (16.7 ​%) between 41 and 60 ​min, and 21 (58.3 ​%) between 61 and 80 ​min.

In terms of the tackle number in the set, there was 1 (2.8 ​%) concussion recorded for tackle zero, 7 (19.4 ​%) concussions that occurred for the first tackle, 9 (25 ​%) that occurred for the second tackle, 4 (11.1 ​%) for the third tackle, 8 (22.2 ​%) concussions for the fourth tackle, 5 (13.9 ​%) for the fifth tackle, and 2 (5.6 ​%) for the sixth tackle.

### 3.2 Video signs of possible concussion

Loss of responsiveness was observed in 22.2 ​% (8/36) of concussions. No protective action was observed in 36.1 ​% (13/36) of concussions. Seizure or tonic posturing was observed in 2.8 ​% (1/36) of concussions. Balance disturbance was observed in 72.2 ​% (26/36) of concussions. A dazed, blank, or vacant stare was observed in 30.6 ​% (11/36) of concussions.

Category II video signs of potential concussion included possible tonic posturing, possible balance disturbance, and slow to stand. Possible tonic posturing was observed in 22.2 ​% (8/36) of concussions. Possible balance disturbance was observed in 97.2 ​% (35/36) of concussions. Slow to stand was observed in 91.7 ​% (33/36) of concussions. In 2 (5.6 ​%) concussions, there were no observable video signs. In 17 (47.2 ​%) concussions there were four or more (out of nine) video signs of potential concussion. For the six international consensus signs [18] only one (2.8 ​%) concussed player displayed four video signs of potential concussion and no concussed player had 5 or 6 video signs of potential concussion.

### 3.3 Return to play

Most players (23, 63.9 ​%) did not miss a game (i.e., they returned to play the following week), while 2 players (5.6 ​%) missed one game, one player (2.8 ​%) missed 3 games, one player (2.8 ​%) missed 4 games, and one player (2.8 ​%) missed 5 games. There were 8 players (22.2 ​%) who did not return to play at this level for the rest of the season (but who may have returned to match play at a lower level of competition). This included players that did not return in 11 weeks (n ​= ​1), 8 weeks (n ​= ​2), 5 weeks (n ​= ​1), 2 weeks (n ​= ​2), and 1 week (n ​= ​1) before the end of the season, and one player who was concussed in his team's final game of the season.

## 4 Discussion

At the sub-elite level of rugby league, during the 2019 men's QRL season, the concussion incidence rate was 6.11 injuries per 1000 player match hours—corresponding to one concussion every 4.7 matches. Nearly two thirds of the players (approximately 64 ​%) returned to play the following game. In other semi-elite rugby league injury surveillance research, concussion incidence rates have generally ranged from 0.4 [20] to 10.5 [21], similar to the results of the current study. However, in one study that reviewed three grades at one club over a single season, the concussion incidence was 34.8 [22]. A pooled analysis of semi-professional rugby league players reported a concussion incidence of 5.9 [23]. The concussion injury rate of male QRL players is lower than data reported at the elite level, where the concussion incidence rate was 8.61, 10.48, and 10.91 concussions per 1000 player match hours during the 2014 [8], 2017, and 2018 [24] NRL season, respectively. This equates to one concussion every 3.4 [8], 2.8, and 2.6 [24] NRL matches. At the NRL level there were approximately 88 ​% of players who did not miss a match following sustaining a concussion in the 2018–2019 seasons [24].

A tackler sustained more concussions than a ball carrier in the QRL. This is consistent with the NRL data that has revealed tacklers are 1.7 times more likely to experience a HIA than a ball carrier [14]. An upright tackler and an upright ball carrier are known to increase the risk for a HIA at the NRL level [14]. In the QRL we observed that 61.1 ​% of concussions occurred with an upright tackler and a ball carrier. Forwards were more likely to be removed from play with a HIA and be medically diagnosed with a concussion than backs in both the QLR and the NRL [12]. In the QRL, 11 ​% of concussions occurred due to foul play compared to the reported 20 ​% in preliminary data from the 2013 NRL season [3].

In the QRL 97.2 ​% (35/36) players diagnosed with concussion had at least one video sign of concussion. The most commonly observed video sign was balance disturbance/motor incoordination, a video sign that was seen in approximately 72 ​% of concussed QRL players. At the NRL level, balance disturbance/motor incoordination from three seasons (2017–2019) was observed in 25.5 ​% of concussed players [7]. Multiple video signs of potential concussion were seen in 44.5 ​% of concussed QRL players. In the NRL there were 62 ​% of concussed players who were considered to have multiple observed video signs [8]. Most studies of concussion video signs from other research groups and in other sports have focused on reporting the frequency of various signs, inter-rater reliability, or the sensitivity, specificity, positive predictive value, and negative predictive value of specific signs and they have used video footage from elite-level/professional leagues [[25], [26], [27], [28], [29], [30]].

### 4.1 Limitations

There are several limitations with the current study. First, there was a limited availability of multiple well-positioned camera angles to view all incidents. This may adversely affect the ability to identify certain game characteristics and video signs of potential concussion (i.e., increase the amount of missing information). Second, the results coded were obtained from one season only, as opposed to multiple seasons. The data from the 2019 season were provided as the most recent season available when the request for data was made to the QRL by the research team. Assimilating the data and video footage was a time-consuming process. After the 2019 season the QRL introduced mandatory stand down periods following a medically diagnosed concussion, and all players have a 10-day non-contact recovery period before they can return to match play. Law changes have included the prohibition of the shoulder charge, and a six-again rule instead of a penalty for ruck infringements. Limiting our study to a single season constrains the potential to use the findings for providing recommendations to reduce concussion risk. Concussion surveillance research from other combat and collision sports [25,26,[31], [32], [33], [34], [35]] evaluated more concussions than the current study [25,26,31,32], over multiple seasons [25,26,31,32,35], with some studies statistically analysing sport-specific characteristics with the aim of providing concussion management recommendations [25,31,32] or sport-specific coaching techniques [34]. For example, numerous concussions in taekwondo were found to result from a roundhouse kick to the temporal region delivered to an opponent in a closed stance position, resulting in recommendations for the development of blocking skills [34]. Third, the training of the coder by an expert was used as a means of establishing reliability and consistency, but two coders did not code all events and therefore inter-rater reliability was not calculated. Past studies examining the inter-rater reliability of video signs of potential concussion of two expert coders have shown good to excellent inter-rater reliability [26] and moderate inter-rater reliability between naïve and expert raters [36]. Fourth, there was no way to verify if a concussed player had returned to match play at a lower competition level. Fifth, the generalizability of the results may not be applicable to other levels of play, women rugby league players, or other age groups.

Understanding how video may aid the decision-making process at the sub-elite level requires further investigation. The extent to which a live feed on the sideline may change the in-game decision-making process at this level is unknown. The current study did not evaluate the clinical profile of the concussed athlete nor the specific duration of time it took for them to return to play. The extent to which video signs may be related to the players’ test scores and results from a medical evaluation, or their recovery and medical clearance to return to play, could be a focus of future work at this level.

## 5 Conclusions

To our knowledge this was one of the first studies to review video footage of concussions in sub-elite rugby league competition. Understanding the differing presentation of video signs and rates of concussion at the sub-elite level compared to the elite level, requires further investigation with larger numbers. Overall, the current findings build on the growing body of video analysis research in rugby league and suggest that the retrospective review of the video of incidents may offer insights into modifiable risk factors (such as ball carrier and tackler pre- and post-contact behaviour) that may help reduce concussion in rugby league.

## Ethics approval and consent to participate

The study was approved by The University of Newcastle's Human Ethics Committee (reference number: H-2015-0323) and was performed in accordance with the standards of ethics outlined in the Declaration of Helsinki. All players gave prior consent to the Rugby League Players Association and the QRL to have their deidentified data used in research endorsed by the QRL Research Committee. This study was approved by the governing institution human ethics committee and allowed by the QRL research committee.

## Consent for publication

Not applicable.

## Availability of data and material

The minimal data set is available in the online supplementary table.

## Authors’ contributions

All authors contributed to the conceptualization and design of this study. MAL and AJG collected the data and created the database for analysis. AJG reviewed and coded a small portion of videos to train MAL, and MAL reviewed all videos and coded the HIA events. AJG provided primary supervision to PhD student MAL and SE provided secondary supervision to PhD student MAL. DPT and GLI conceptualised and conducted the statistical analysis. All authors contributed to the drafting of portions of the manuscript, critically reviewed manuscript drafts, and read and approved the submission of the final version. All authors agree to be accountable for all aspects of the work.

## Funding

There was no direct or sponsored funding for this study. Martin A. Lang acknowledges support from the University of Newcastle Higher Degree Research Funding Scheme.

## Declaration of competing interest

Martin A. Lang has nothing to declare.

Grant Iverson, Ph.D. has been reimbursed by the government, professional scientific bodies, and commercial organizations for discussing or presenting research relating to mild TBI and sport-related concussion at meetings, scientific conferences, and symposiums. He has a clinical and consulting practice in forensic neuropsychology, including expert testimony, involving individuals who have sustained mild TBIs (including athletes). He has received research funding from several test publishing companies, including ImPACT Applications, Inc., CNS Vital Signs, and Psychological Assessment Resources (PAR, Inc.). He has received research funding from the National Football League. He has also received research funding from the Harvard Integrated Program to Protect and Improve the Health of National Football League Players Association Members.

Suzi Edwards leads a tackle re-education program that is partially finded by a 2021 NHMRC Ideas Grant (202718).

Ben Jones, PhD, is employed by Leeds Rhinos, Rugby Football League and Premiership Rugby in a consultancy capacity.

Douglas Terry, PhD., serves as a scientific advisor for HitIQ. He previously consulted for REACT Neuro, Inc. He has a consulting practice in forensic neuropsychology, including expert testimony, involving individuals who have sustained mild TBIs (including former athletes). He received research funding from Amgen, Inc. and Football Research Inc.

Andrew Gardner, Ph.D. has a clinical practice in neuropsychology involving individuals who have sustained sport-related concussion. He is a contracted concussion consultant to Rugby Australia. He is the global clinical lead for the World Rugby Brain Health Service. He is a member of the World Rugby Concussion Working Group, and a member of the Australian Football League Concussion Scientific Advisory Committee. He has received travel funding or been reimbursed by professional sporting bodies, and commercial organisations for discussing or presenting sport-related concussion research at meetings, scientific conferences, workshops, and symposiums. Previous grant funding includes the NSW Sporting Injuries Committee, the Brain Foundation (Australia), an Australian-American Fulbright Commission Postdoctoral Award, a Hunter New England Local Health District, Research, Innovation and Partnerships Health Research & Translation Centre and Clinical Research Fellowship Scheme, and the Hunter Medical Research Institute (HMRI), supported by Jennie Thomas, and the HMRI, supported by Anne Greaves. He is currently supported by a National Health and Medical Research Council (NHMRC) Investigator Grant. He acknowledges unrestricted philanthropic support from the Tooth Foundation for concussion research and the National Rugby League for research in retired professional rugby league players.
