# Supplementary material for: Examining concussions in adult male, senior-grade semi-elite rugby league in Australia: A retrospective observational video review case series
Source: JSAMS Plus. 2025 Jan 2;5:100086. doi: 10.1016/j.jsampl.2024.100086 (PMC13008426; doi:10.1016/j.jsampl.2024.100086)
Supplement: Multimedia component 1 [file mmc1.docx]

**Supplementary Table. Video signs for the 36 concussion cases.**

| Study  No. | Video Signs | | | | | | | | | | | Ball Carrier  v  Tackler | Playing Position | Tackler  Body  Position | Ball Carrier  Body  Position | Tackler Contact  Height | Head  Contact  From | Foul  Play | Tackle  No. in Set |
| --- | --- | --- | --- | --- | --- | --- | --- | --- | --- | --- | --- | --- | --- | --- | --- | --- | --- | --- | --- |
|  | LOC | No  Protective  Action | Seizure | Tonic  Posturing | Balance  Disturbance | Dazed or  BL or VS | Loss of  Responsiveness | Possible  Tonic  Posturing | Possible  Balance  Disturbance | Slow To  Stand | Total  Video  Signs |  |  |  |  |  |  |  |  |
| 1 | No | No | No | No | Yes | No | No | No | Yes | Yes | 3 | T | Fwd | BAW | Upright | MT | Elbow | 0 | 4 |
| 2 | No | No | No | No | Yes | No | No | No | Yes | Yes | 3 | T | Fwd | Upright | Upright | UT | Head | 0 | 2 |
| 3 | No | Yes | No | No | Yes | No | No | No | Yes | Yes | 4 | Neither | Fwd | Flopping | On ground | Head | Ground | 0 | 4 |
| 4 | Yes | Yes | No | No | Yes | Yes | Yes | Yes | Yes | Yes | 8 | T | Bk | Leap/jump | Upright | LL | Knee | 0 | 0 |
| 5 | Yes | Yes | No | No | Yes | Yes | Yes | Yes | Yes | Yes | 8 | T | Bk | Diving | Fall/Dive | LT | Ground | 0 | 6 |
| 6 | No | No | No | No | No | No | No | No | Yes | No | 1 | Neither | Fwd | Upright | BAW | UT | Head | 0 | 4 |
| 7 | Yes | Yes | No | No | Yes | Yes | Yes | Yes | Yes | Yes | 8 | T | Bk | Upright | Upright | UT | Forearm | 0 | 3 |
| 8 | No | No | No | No | Yes | No | No | No | Yes | Yes | 3 | T | Fwd | Upright | Upright | UT | Head | 0 | 5 |
| 9 | No | Yes | No | No | Yes | No | No | Yes | Yes | Yes | 5 | T | Bk | Upright | BAK | UT | Head | 0 | 6 |
| 10 | No | No | No | No | Yes | No | No | No | Yes | Yes | 3 | T | Bk | Leap/jump | Upright | UT | Head | 0 | 4 |
| 11 | No | Yes | No | No | Yes | No | No | No | Yes | Yes | 4 | BC | Fwd | Upright | Upright | UT | Ground | 0 | 5 |
| 12 | No | No | No | No | Yes | Yes | No | No | Yes | Yes | 4 | BC | Fwd | Upright | Upright | Head | Forearm | 1 | 2 |
| 13 | No | No | No | No | No | No | No | No | No | No | 0 | T | Fwd | Upright | Upright | UT | Unknown | 0 | 2 |
| 14 | No | No | No | No | Yes | No | No | No | Yes | Yes | 3 | BC | Fwd | Upright | BAW | UT | Unknown | 0 | 2 |
| 15 | No | No | No | No | No | No | No | No | Yes | Yes | 2 | T | Bk | Upright | Upright | UT | Forearm | 0 | 4 |
| 16 | No | No | No | No | Yes | No | No | No | Yes | Yes | 3 | BC | Fwd | Upright | Upright | UT | Head | 0 | 1 |
| 17 | Yes | Yes | No | No | Yes | Yes | Yes | Yes | Yes | Yes | 8 | T | Fwd | Upright | Upright | UT | Head | 0 | 1 |
| 18 | No | Yes | No | No | No | No | No | No | Yes | Yes | 3 | BC | Bk | Upright | Upright | UT | Shoulder | 0 | 3 |
| 19 | No | No | No | No | Yes | No | No | No | Yes | Yes | 3 | T | Bk | Diving | Upright | LT | Knee | 0 | 2 |
| 20 | Yes | No | No | No | Yes | No | Yes | Yes | Yes | Yes | 6 | T | Fwd | Upright | Upright | UT | Head | 0 | 1 |
| 21 | Yes | Yes | No | No | No | Yes | Yes | Yes | Yes | Yes | 7 | T | Bk | Upright | Upright | UT | Head | 0 | 4 |
| 22 | Yes | Yes | Yes | No | Yes | Yes | Yes | Yes | Yes | Yes | 9 | T | Fwd | Upright | Upright | UT | Shoulder | 0 | 1 |
| 23 | No | Yes | No | No | Yes | No | No | No | Yes | Yes | 4 | BC | Bk | Upright | Upright | Head | Shoulder | 1 | 4 |
| 24 | No | No | No | No | Yes | Yes | No | No | Yes | Yes | 4 | BC | Fwd | Upright | Upright | Head | Forearm | 1 | 1 |
| 25 | No | No | No | No | Yes | Yes | No | No | Yes | Yes | 4 | T | Fwd | Upright | Upright | UT | Forearm | 0 | 3 |
| 26 | No | Yes | No | No | Yes | No | No | No | Yes | Yes | 4 | T | Fwd | Diving | Upright | MT | Buttocks | 0 | 5 |
| 27 | No | No | No | No | No | No | No | No | Yes | Yes | 2 | T | Fwd | BAW | Upright | MT | Hip | 0 | 4 |
| 28 | No | No | No | No | Yes | Yes | No | No | Yes | Yes | 4 | T | Fwd | Upright | Upright | UT | Head | 0 | 1 |
| 29 | No | No | No | No | No | No | No | No | Yes | No | 1 | T | Fwd | Upright | Upright | UT | Head | 0 | 2 |
| 30 | No | No | No | No | No | No | No | No | Yes | Yes | 2 | T | Fwd | BAW | Fall/Dive | UT | Head | 0 | 2 |
| 31 | No | No | No | No | Yes | No | No | No | Yes | Yes | 3 | BC | Fwd | Leap/jump | Upright | Head | Arm | 1 | 1 |
| 32 | No | No | No | No | Yes | No | No | No | Yes | Yes | 3 | T | Fwd | Upright | Upright | UT | Head | 0 | 3 |
| 33 | No | No | No | No | Yes | No | No | No | Yes | Yes | 3 | BC | Fwd | BAW | Upright | MT | Ground | 0 | 5 |
| 34 | No | No | No | No | No | No | No | No | Yes | Yes | 2 | T | Fwd | Upright | Upright | UT | Shoulder | 0 | 2 |
| 35 | No | No | No | No | No | No | No | No | Yes | Yes | 2 | T | Fwd | Upright | Upright | UT | Shoulder | 0 | 2 |
| 36 | Yes | Yes | No | No | Yes | Yes | Yes | No | Yes | Yes | 7 | BC | Bk | Upright | Upright | UT | Shoulder | 0 | 5 |

Note. BAK: bent at knees; BAW: bent at waist; BC: ball carrier; Bk: back; BL: blank look; Fwd: forward; MT: mid trunk; LL: lower leg; LOC: loss of consciousness; LT: lower trunk; NA: not applicable; No.: number; T: tackler; UT: upper trunk; v: versus; VS: vacant stare.
